# A comparison between knotted and knotless medial row of suture bridge technique in arthroscopic rotator cuff repair surgery: a meta-analysis

**DOI:** 10.1186/s13018-023-03812-7

**Published:** 2023-05-08

**Authors:** Qiuping Xiao, Xiaolin Quan, Shidong Hu, Yujia Xiao, Jiangping Wu, Mao Nie

**Affiliations:** 1 Center for Joint Surgery, Department of Orthopedic Surgery, The Second Affiliated Hospital of Chongqing Medical University, Chongqing, China; 2 Chongqing Nanchuan District People’s Hospital, Chongqing, China

**Keywords:** Suture bridges, Double-row suture bridge techniques, Knotted, Knotless, Retear rate

## Abstract

**Background:**

The shoulder arthroscopic suture bridge technique is currently very popular, but scientific evidence relating to the clinical outcomes of the medial row with or without knots has not been systematic reviewed.

**Purpose:**

The purpose of this study was to compare the clinical outcomes of knotted versus knotless double-row suture bridges for rotator cuff repairs.

**Study design:**

Meta-analysis.

**Method:**

Five databases that contain literature in English were searched (Medline, PubMed, Embase, Web of Science, and the Cochrane Library), with a focus on works published between 2011 and 2022. Clinical data relating to arthroscopic rotator cuff repair with the suture bridge approach was examined and the outcomes of medial row knotting contrasted with that of the knotless technique. The search phrase used was: *(double row) AND (rotator cuff) AND (repair),* and the search method is subject term plus free word search. Literature quality evaluation was performed using the Cochrane “risk of bias” tool 1.0 and the Newcastle–Ottawa scale quality assessment instrument.

**Results:**

One randomized controlled trial, four prospective cohort studies, and five retrospective cohort studies were included in this meta-analysis. Data pertaining to 1146 patients was drawn from these ten original papers and analyzed. Meta-analyses that were performed on 11 postoperative outcomes revealed that none of the differences were statistically significant (*P* > 0.05) and that the publications were unbiased (*P* > 0.05). Postoperative retear rate and postoperative retear categorization were the outcomes assessed. Scores on postoperative pain, forward flexion, abduction, and external rotation mobility were collated and evaluated. The University of California, Los Angeles scoring systems in the first year following surgery, the American Shoulder and Elbow Surgeons score and Constant scales in the first and second years after surgery were the secondary outcomes spotlighted in this study.

**Conclusion:**

The clinical outcomes of shoulder arthroscopic rotator cuff repair with the suture bridge technique with or without a knotted medial row was proven to be equivalent. These outcomes are about postoperative retear, postoperative retear classification, postoperative shoulder function score, postoperative shoulder mobility, and postoperative pain, respectively. It should be noted that the conclusions are based on short-term clinical follow-up data.

*What is known about this subject* The shoulder arthroscopic suture bridge technique is currently very popular, but scientific evidence relating to the clinical outcomes of the medial row with or without knots has not been systematic reviewed. Both techniques can achieve good clinical results.

*What this study adds to existing knowledge* The clinical outcomes of shoulder arthroscopy rotator cuff repair with the suture bridge technique with or without a knotted medial row was proven to be equivalent. These outcomes are about postoperative retear, postoperative retear classification, postoperative shoulder function score, postoperative shoulder mobility, and postoperative pain, respectively. Nevertheless, the clinical outcomes of the knotless Mason-Allen suture bridge technique needs to be explored in larger sample sizes in future studies.

## Introduction

Rotator cuff tears are a common, musculoskeletal injury accounting for many of the surgeries performed in the ambulatory setting [18]. Arthroscopic rotator cuff repair is now widely performed and has become the first-line treatment for rotator cuff injuries.

Various arthroscopic suture repair techniques have been developed to date. The suture bridge technique, as one type of double-row suture technique, is currently preferred by many orthopedic surgeons for its improved contact area, increased pressure load, and reduced operating time [9, 29]. Even though there are various suture designs, suture bridge methods are commonly described as either having a knotted medial row of suture anchors or having a knotless medial row of suture anchors. In traditional suture wire methods, a knotted medial row is combined with a knotless lateral row to create a knotless structure. On the other hand, the knotless suture bridge technique uses a high-strength flat-braided suture-type that is resistant to pull-through [2].

Numerous biomechanical investigations have contrasted rotator cuff restoration procedures using knotted and knotless suture bridges. An experiment performed by Busfield and his colleague [4] revealed that the addition of a knotless medial row could compromise the construct leading to gap formation and failure at lower loads. Furthermore, Maxwell et al. [31] observed that knotless repair might exhibit an enhanced self-reinforcing effect, without reducing footprint contact, a benefit that medial knotting is unable to confer. In terms of displacement across the repair site, stiffness, and ultimate load to failure, Mijares et al. [25] discovered that knotted and knotless medial-row double-row rotator cuff repair structures using suture tape showed similar biochemical performance. High level scientific evidence has demonstrated that suture bridge sutures with medial row knots have better biomechanical effects, including greater ultimate load, and no significant differences in terms of suture formation, suture stability, or footprint contact area [1].

Conflicting results have arisen from clinical studies investigating rotator cuff suture bridge repair with knotted versus knotless medial rows. A case–control study by Hirokazu and his colleagues [17] found that incomplete healing at the 24-month mark post-surgery was more common among patients with medial row knots. However, according to Kyung et al. [20], even though the knotless suture-bridge technique had a higher rate of retear than the standard suture-bridge technique, the difference was not statistically significant. Boyer et al. [3] found that the retear rate for the knotless tape-bridging construct was lower but not significantly. In a tendon repaired using a knot-tying method, Şahin et al. [34] found that this procedure increased the risk of failure at the medial musculotendinous junction, with a laterally healed tendon on the footprint. According to Cho’s classification [6], postoperative retear can be divided into 2 types: type 1 retear is defined as separation at the footprint of a repaired rotator cuff, while failure at the medial musculotendinous junction is the definition of a type 2 retear, which occurs when there is a laterally healed tendon on the footprint.

To date, a scientific review of this topic is absent in past and current literature. The authors wish to review the key literature, extract key data from it, and use the data to assess the clinical outcomes of the knotted vs knotless double-row suture bridge approaches for rotator cuff surgery.

## Methods

The PRISMA (Preferred Reporting Items for Systematic Reviews and Meta-Analyses) declaration has been followed throughout this publication [26]. PROSPERO has been used to create and register the protocol for this publication (CRD42022357604).

### Inclusion and exclusion criteria

Only the clinical publications comparing medial rows of suture bridges for repairing torn rotator cuff under shoulder arthroscopy with knotted versus knotless knots were be included.

The following were among the criteria for inclusion:

- *Patient* Regardless of age, gender, illness course, comorbidity, or other variables across different groups in the same study, studies involving patients who suffered rotator cuff injuries in the past and underwent shoulder arthroscopic surgery were included.
- *Experimental Design* Studies that contrasted the clinical outcomes of rotator cuff repair methods using knotted and knotless suture bridges were included.
- *Outcome Measures* Studies whose main outcomes were postoperative retear rate and postoperative retear categorization were included. The following were the secondary outcomes of interest: scores on postoperative pain, forward flexion, abduction, and external rotation mobility, as well as the University of California, Los Angeles (UCLA) scoring systems in the first year following surgery, the American Shoulder and Elbow Surgeons (ASES) score and Constant scales in the first and second years after surgery were the secondary outcomes.
- *Study Design* Randomized clinical trial comparing knotted rotator cuff repair suture bridges and knotless suture bridges during shoulder arthroscopy was included.

The following studies were excluded: biomechanical studies, cadaveric studies, and non-clinical studies of rotator cuff injury suture type. The following publications were also excluded: newspapers, unreviewed articles, case reports, meta-analyses, reviews, opinion pieces, anecdotal studies, case studies involving < 10 cases, editorials, comments, book chapters, and conference proceedings or abstracts.

### Literature search strategy

The Cochrane Library, Medline, Embase, Web of Science, and Pubmed were all thoroughly searched. Using the keywords "double row, rotator cuff, repair," studies published from 2010 to the present were identified. The aforementioned keywords were combined with their free words to search. The legitimacy of candidate publications was assessed using the aforementioned standards.

### Data extraction and synthesis

Data was extracted and recorded on electronic spreadsheets from eligible studies by two independent reviewers. Through discussion, a consensus was reached and differences in inclusion were preliminarily resolved. In case of further disagreement, the final decision was made by the third reviewer. Author(s), country, publication year, research focus, research design, classification, patient/case count (male and female), functional scores, number of retear following surgery, postoperative retear classification, records of a postoperative range of motion, and postoperative pain scores were all extracted from the study. Whenever possible, the missing data were sourced from the relevant corresponding authors included in the study.

### Quality assessment

Independently, two reviewers evaluated the risk of bias. The deviation risk instrument (Version 1.0; Cochrane Collaboration) [10] was employed: to assess the potential deviation of each test. This tool made it possible to divide the deviation into seven distinct categories, which were as follows: selection deviation; allocation concealment deviation; blinding deviation; result reporting deviation; outcome completeness deviation; and other categories. Each element was divided into three risk categories: low risk, medium risk, and high risk. Case selection, comparability, and outcome reporting were evaluated using the Newcastle–Ottawa scale (NOS) [35], a tool for evaluating the quality of cohort studies included in a systematic review and/or meta-analyses.

### Data analysis

Meta-analysis statistics and the creation of forest plot data were performed using Stata SE 15.0 (StataCorp, USA). Heterogeneity was also calculated using *I*^2^ statistics and the Q test [11]. The mean difference (MD) and 95% confidence interval (CI) for continuous data were calculated, the risk ratio (RR) value for non-continuous data determined and a forest plot generated to visually illustrate the data. In the event that there was evidence of heterogeneity after the execution of the first analyses using a fixed-effects model, the random-effects regression model was considered for use in the subsequent meta-analysis. *P* values below 0.05 were considered to be statistically significant. Finally, a test for publication bias was conducted using the Begg’s method. *P* values above 0.05 were an indicator of low publication bias [11].

## Results

### Literature search

**Fig. 1:**
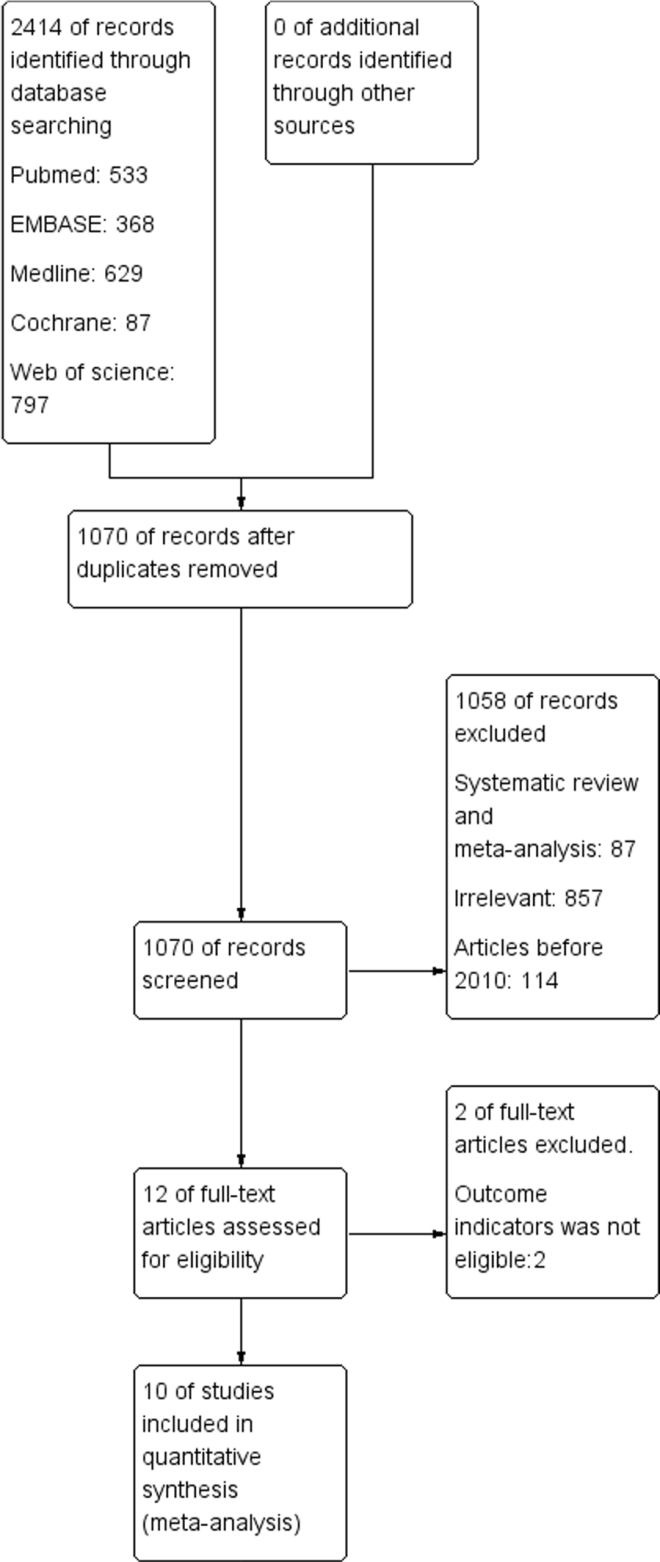
PRISMA flowchart of study selection

A total of 2414 potentially relevant publications were found using the first search keyword (Pubmed: 533; Medline: 437; Web of Science: 797; Embase: 368; Cochrane Library: 87). Each publication was reviewed, and 10 articles selected for inclusion in the meta-analysis (Fig. 1).

### Characteristics of selected studies

There were ten studies [3, 15–17, 20, 28, 33, 34, 37, 40] that compared arthroscopic double-row suture bridge repair of the rotator cuff with and without knotting of the medial row, published between 2011 and 2022. One of the included studies was a prospective randomized controlled trial, four of the studies were prospective cohort trials, and the other five studies were retrospective cohort trials. A total of 1146 participants were included in this analysis. In terms of clinical outcomes, nine studies focused on postoperative retear rate, while six focused on postoperative retear classification. Nine studies reported functional score results, three of which described UCLA scores in the first year postoperatively, two described ASES scores in the same, two described Constant scores in the same, five described Constant scores in the second year, three described ASES scores in the second year. For other outcomes, six articles reported postoperative mobility, five of which reported postoperative anterior flexion mobility, three reported postoperative abduction mobility, and three reported postoperative external rotation mobility. In the context of pain scores, four articles reported on postoperative visual pain scores. For the suture techniques, seven out of ten articles compared the traditional suture-bridge repair with and without medial knot tying. Of the other three articles, one article compared double-pulley suture-bridge repair with and without medial knot tying, one compared the traditional suture-bridge technique with knotted medial row and knotless double-layer transosseous equivalent repair technique (cinch-bridge), and one compared traditional knot-tying suture bridge technique and knotless medial row suture-bridge technique using Mason-Allen stich for medial row fixation. For the type of rotator cuff retraction, there were five articles that mentioned the type of rotator cuff retraction, and there was no significant difference between the knotted and knotless groups in these five articles. Similarly, there were seven articles that mentioned the grade of rotator cuff fatty infiltration, and there were no significant differences between the knotted and knotless groups. For the remaining baseline indicators such as gender and age, there were no significant differences between the knotted and knotless groups for each study (Tables 1 and 2).

**Table 1:** Characteristics of the included studies

References	Design	Patients' diagnosis	Intervention	Sample size (knotted group/knotless group, *n*)	Age [years, knotted group/knotless group, M ± SD or mean (range)]	Gender (knotted group/knotless group, male/female)	Method/technique of fixation stich	Rotator cuff retraction pattern (**Patte classification)	Rotator cuff fatty infiltration (***Goutallier classification)
Hirokazu et al. [17]	Retrospective CT	Full-thickness cuff tears	Knotted group /knotless group	29/24	63.8 ± 8.4/65.1 ± 9.6	(17/12)/(15/9)	Traditional suture-bridge repair: knotted method versus knotless method	Not mentioned	Not mentioned
Şahin et al. [34]	Prospective RCT	Full-thickness rotator cuff tears	Knotted group/knotless group	42/46	54.3 ± 9.8/55.8 ± 8.2	(12/30)/(20/26)	Traditional suture-bridge repair: knotted method versus knotless method	1, 2, 3	0, 1, 2, 3
Xu et al. [37]	Retrospective CT	*Large full-thickness rotator cuff tears	Knotted group/knotless group	158/134	65.6 ± 8.9/63.9 ± 9.1	(65/93)/(47/87)	Double-pulley suture-bridge repair: knotted method versus knotless method	Not mentioned	0, 1, 2
Zwolak et al. [40]	Retrospective CT	Full-thickness rotator cuff tears	Knotted group/knotless group	64/19	61(42–75)/65(52–81)	(35/29)/(11/8)	Traditional suture-bridge repair: knotted method versus knotless method	1, 2, 3	1, 2, 3
Boyer et al. [3]	Prospective CT	Full-thickness supraspinatus tears	Knotted group/knotless group	38/35	58.8 ± 6.3/57.5 ± 6	(22/16)/(21/14)	Traditional suture-bridge repair: knotted method versus knotless method	1, 2, 3	0, 1
Kim et al. [20]	Prospective CT	Full-thickness supraspinatus or infraspinatus tears 1–4 cm in length in the anterior-to-posterior dimension	Knotted group/knotless group	50/50	59.4 ± 7.45/59.9 ± 7.66	(28/22)/(24/26)	Traditional suture-bridge repair: knotted method versus knotless method	1, 2	Not mentioned
Gürpınar et al. [15]	Prospective CT	*Medium- (1–3 cm) and large-sized tears (3–5 cm)	Knotted group/knotless group	64/57	56.7 ± 7.7/56.6 ± 7.0	(32/32)/(23/34)	Traditional suture-bridge repair: knotted method versus knotless method	1, 2	0, 1, 2
Nemirov et al. [28]	Retrospective CT	Rotator cuff tears	Knotted group/knotless group	117/72	59.2 ± 8.5/55.1 ± 8.6	(82/35)/(38/34)	Traditional suture-bridge repair: knotted method versus knotless method	Not mentioned	Not mentioned
Heuberer et al. [16]	Prospective CT	Full-tendon full-thickness supraspinatus tears and partial tendon full-thickness infraspinatus tears (2.0–3.5 cm)	Knotted group/knotless group	20/17	64.8 ± 7.7/62.8 ± 9.8	(10/10)/(5/12)	Traditional suture-bridge technique with knotted medial row versus knotless double-layer transosseous equivalent repair technique (cinch-bridge)	Not mentioned	0, 1, 2, 3
Rhee et al. [33]	Retrospective CT	*Medium-sized tears	Knotted group/knotless group	59/51	57.6 (45–70) /61.0 (44–68)	(30/29)/(30/21)	Traditional suture-bridge technique with knotted medial row versus knotless medial row suture-bridge technique using Mason-Allen stich for medial row fixation	Not mentioned	Not mentioned

*RCT* randomized controlled trial, *CT* cohort trial, *M* ± *SD* mean ± standard deviation

*According to the classification of DeOrio [12], tear size was categorized as small (< 1 cm), medium (1–3 cm), large (3–5 cm), or massive (> 5 cm)

**Classification of rotator cuff lesions according to the classification of Patte [32]

***Classification of fatty infiltration of the rotator cuff [14]

**Table 2:** Characteristics of the included studies

References	Outcome (Tick in the box if the indicator is available)										
	Postoperative retear rate	Postoperative retear classification	UCLA score in the first year after surgery	ASES score in the first year after surgery	ASES score in the second year after surgery	Constant score in the first year after surgery	Constant score in the second year after surgery	Postoperative Forward flexion mobility	Postoperative abduction mobility	Postoperative external rotation mobility	Postoperative pain score
Hirokazu et al. [17]	√	√	√								
Şahin et al. [34]	√	√						√	√	√	√
Xu et al. [37]	√	√	√	√		√					
Zwolak et al. [40]								√	√	√	
Boyer et al. [3]	√	√					√	√			
Kim et al. [20]	√	√	√	√	√	√	√				√
Gürpınar et al. [15]	√						√	√			√
Nemirov et al. [28]					√						√
Heuberer et al. [16]	√				√		√				
Rhee et al. [33]	√	√									

*UCLA* University of California, Los Angeles, *ASES* American Shoulder and Elbow Surgeons

### Quality assessment

**Fig. 2:**
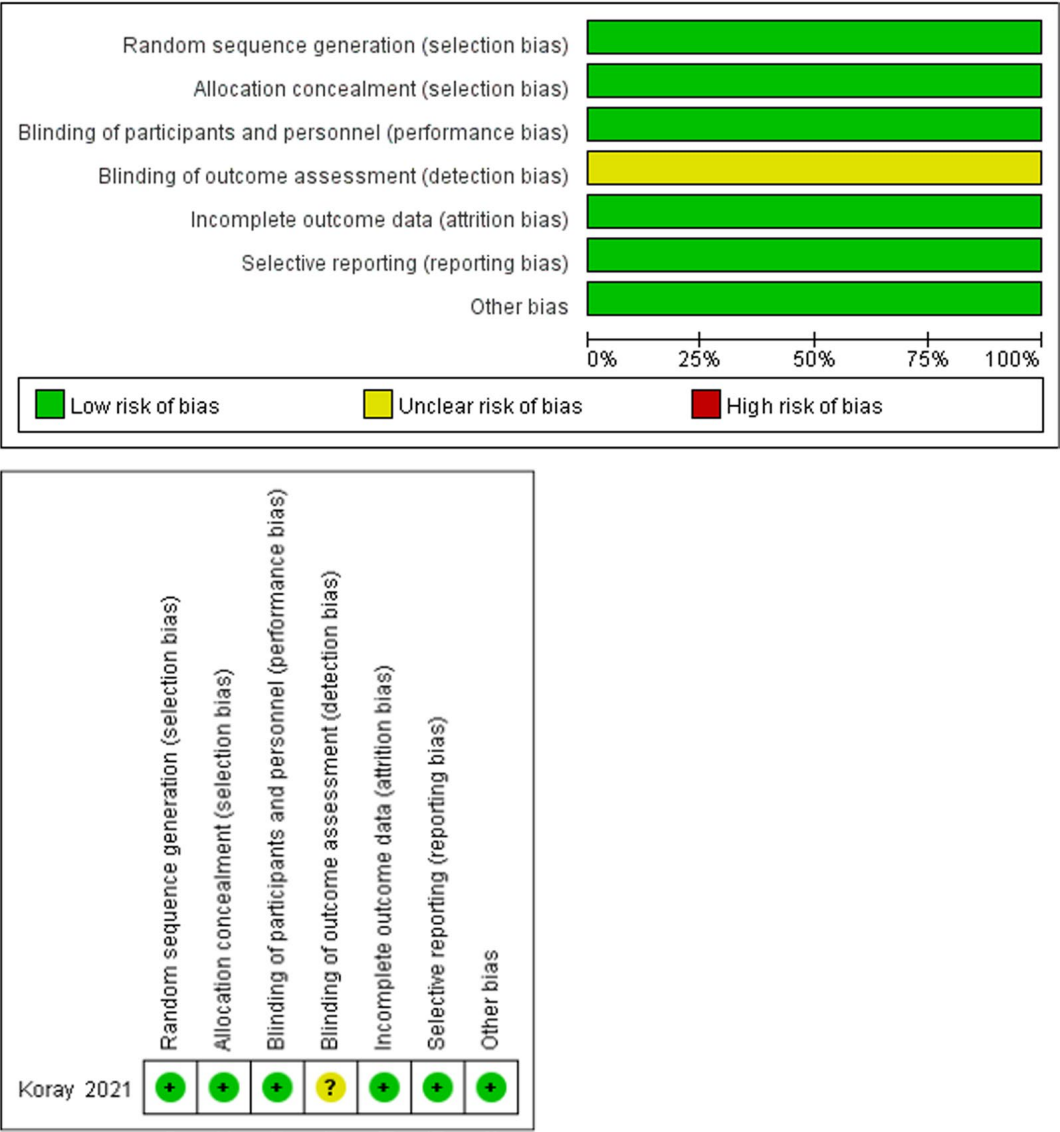
Quality assessment of RCT

Sources of bias in the selected randomized controlled trials (RCT) were assessed using the Cochrane risk assessment tool. Only one study was determined to carry medium risk blinded to the outcome assessor, while the remaining items exhibited low risk. The quality of the remaining nine cohort studies was assessed using the NOS scale, and the results showed that all nine studies attained more than seven stars with no significant risk bias. Two of these studies achieved nine stars, the highest quality, and five achieved eight stars, meaning that the quality of the five was satisfactory. The results are displayed in Fig. 2 and Table 3.

**Table 3:** Quality assessment of cohort trials

References	Selection	Comparability	Outcome	Total
Hirokazu et al. [17]	★★★★	★★	★★★	9
Xu et al. [37]	★★★★	★	★★★	8
Zwolak et al. [40]	★★★★	★	★★★	8
Boyer et al. [3]	★★★★	★★	★★	8
Kim et al. [20]	★★★★	★	★★	7
Gürpınar et al. [15]	★★★★	★★	★★★	9
Nemirov et al. [28]	★★★★	★★	★★	8
Heuberer et al. [16]	★★★★	★	★★	7
Rhee et al. [33]	★★★★	★★	★★	8

### Meta-analysis

#### Postoperative retear rate

**Fig. 3:**
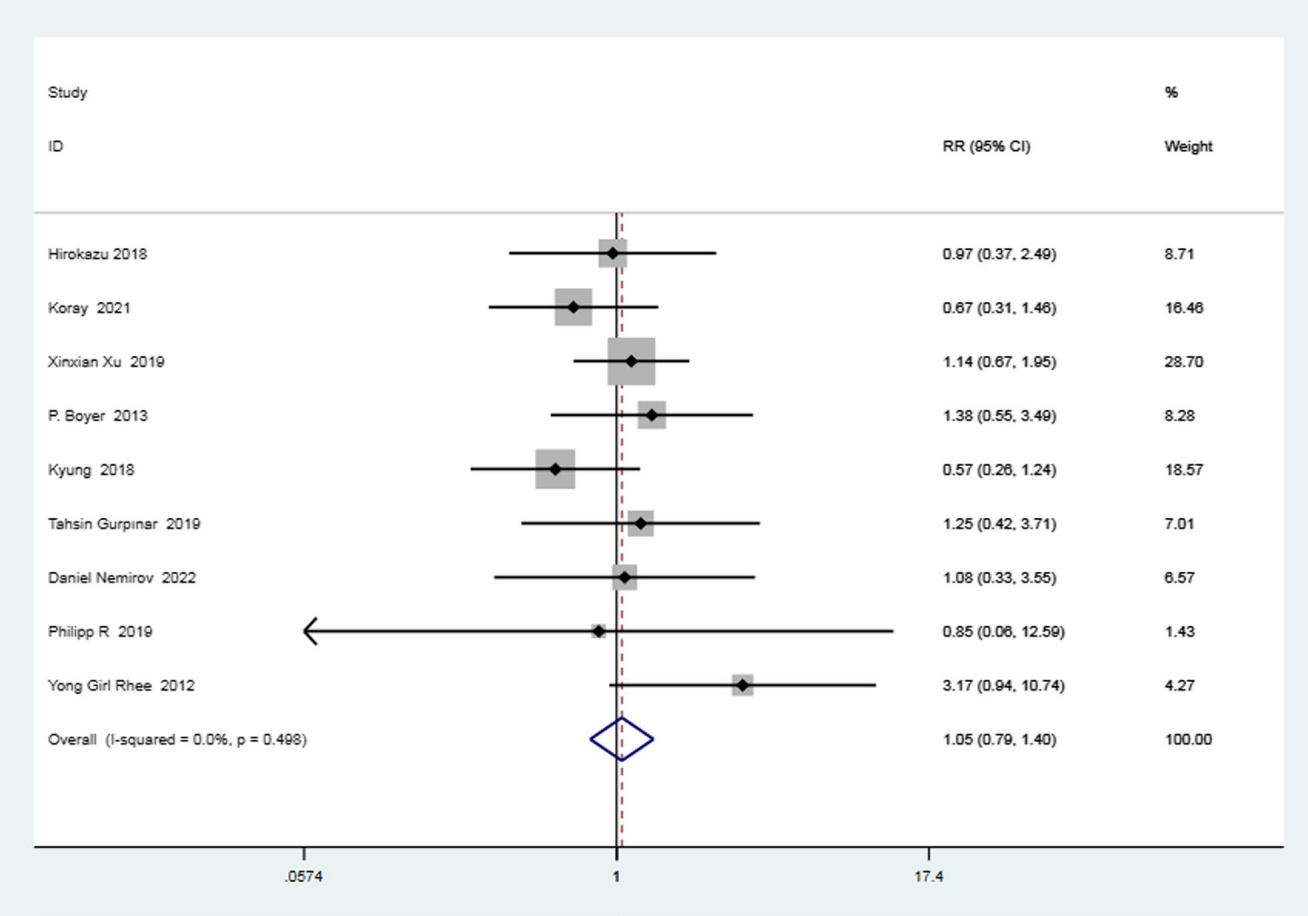
Forest plot of postoperative retear rate. *Note* RR = retear rate of knotted group/retear rate of knotless group

Nine studies [3, 15–17, 20, 28, 33, 34, 37] reported on postoperative retear rate. The heterogeneity test was performed, and the result indicated slight statistical heterogeneity (*I*^2^ = 0 < 50%, *P* = 0.50 > 0.1). The result of meta-analysis showed that the knotted group's retear rate was 1.05 times higher than that of the knotless group, with the difference being statistically insignificant (RR = 1.05, the 95%CI 0.79–1.40, *P* = 0.74 > 0.05). The data are illustrated in the following forest plot (Fig. 3).

#### Postoperative retear classification

**Fig. 4:**
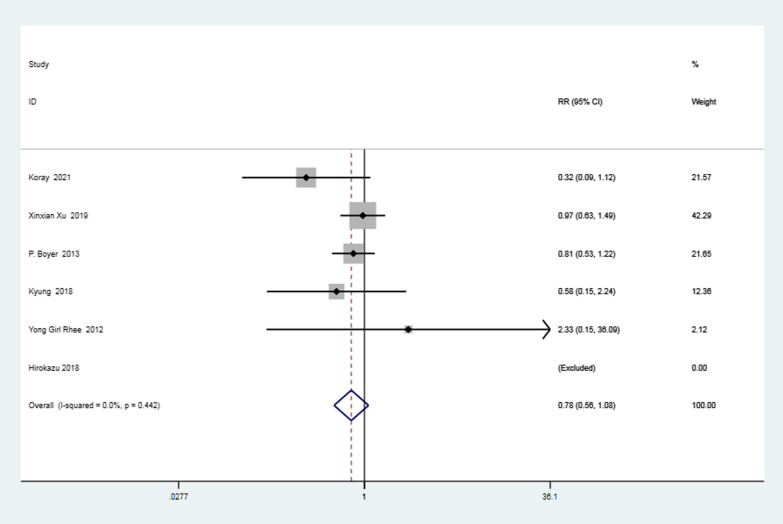
Forest plot of postoperative retear classification. *Note* RR = type 1 retear rate of knotted group/type 1 retear rate of knotless group

Six articles [3, 17, 20, 33, 34, 37] assessed the results of postoperative retear within each class. Firstly, a heterogeneity test was performed, and the result showed that there was no heterogeneity between these studies (*I*^2^ = 0% < 50%, *P* = 0.44 > 0.1). A meta-analysis indicated that the ratio of type 1 retear in the knotted group to those in the knotless group was 0.78, denoting a decreased frequency of type 1 retear in the knotted group though statistically insignificant (RR = 0.78, 95%CI, 0.56–1.08, *P* = 0.14 > 0.05). The data is displayed in the following forest plot (Fig. 4).

#### UCLA score in the first postoperative year

**Fig. 5:**
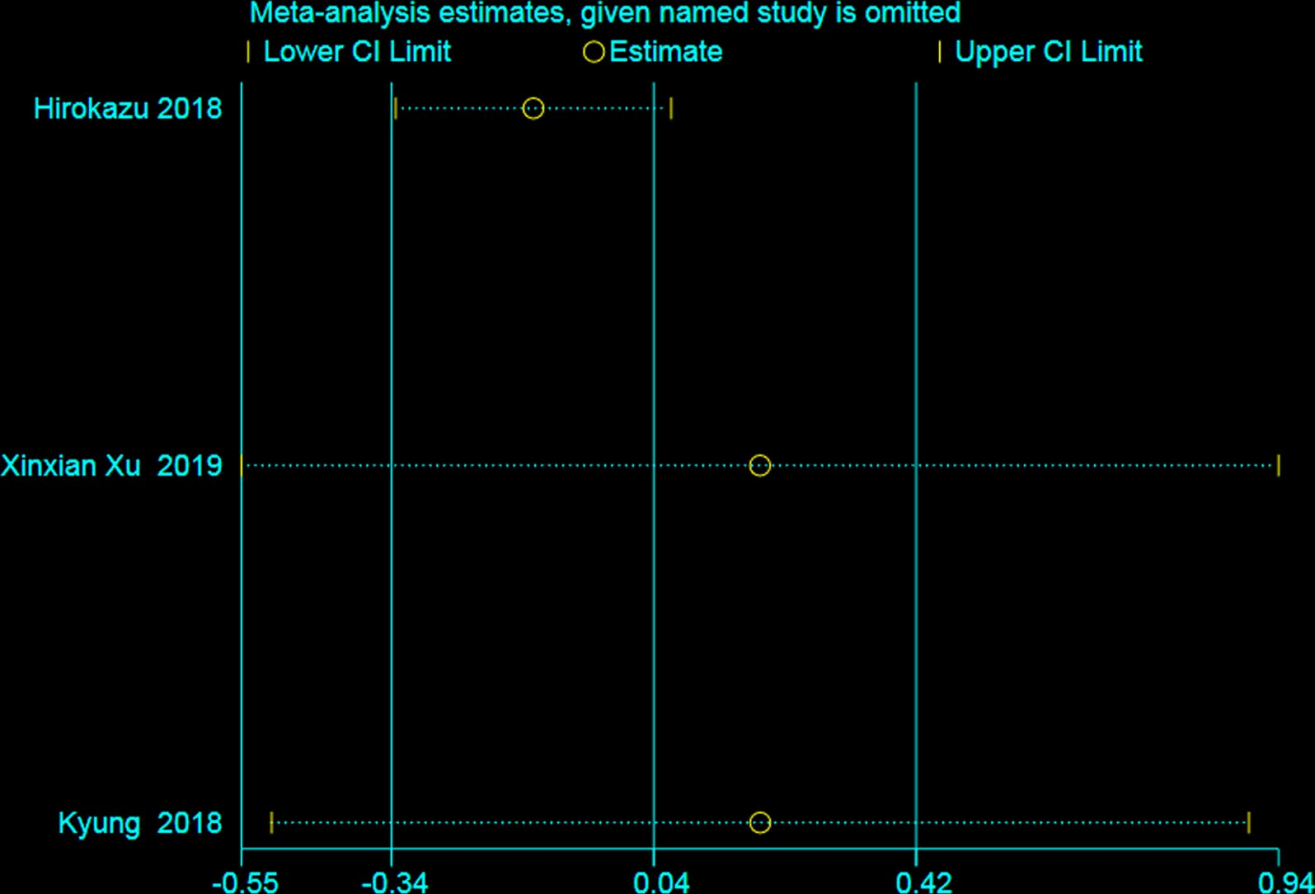
Sensitivity analysis of UCLA score in the first year after surgery

The three studies [17, 20, 37] which reported the UCLA score in the first year after surgery were subjected to a heterogeneity test, which revealed that the studies were homogenous enough to combine (*I*^2^ = 66.8% > 50%, *P* = 0.05 < 0.1). Heterogeneity in the studies was further probed using sensitivity analysis, which confirmed the robustness of the studies. The results are displayed in Fig. 5. However, the degree of variation in the study performed by Hirokazu and his colleagues [17] differed significantly from the other two studies. The heterogeneity test was performed again after excluding this study revealed that the other two studies were highly homogenous (*I*^2^ = 0% < 50%, *P* = 0.98 > 0.1). The meta-analysis revealed that the postoperative UCLA score assigned to the knotless group was 0.59 points higher than that assigned to the knotted group, though the difference was statistically insignificant (MD =  − 0.59, 95%CI − 1.48 to 0.29, *P* = 0.19 > 0.05).

### ASES score in the first postoperative year

**Fig. 6:**
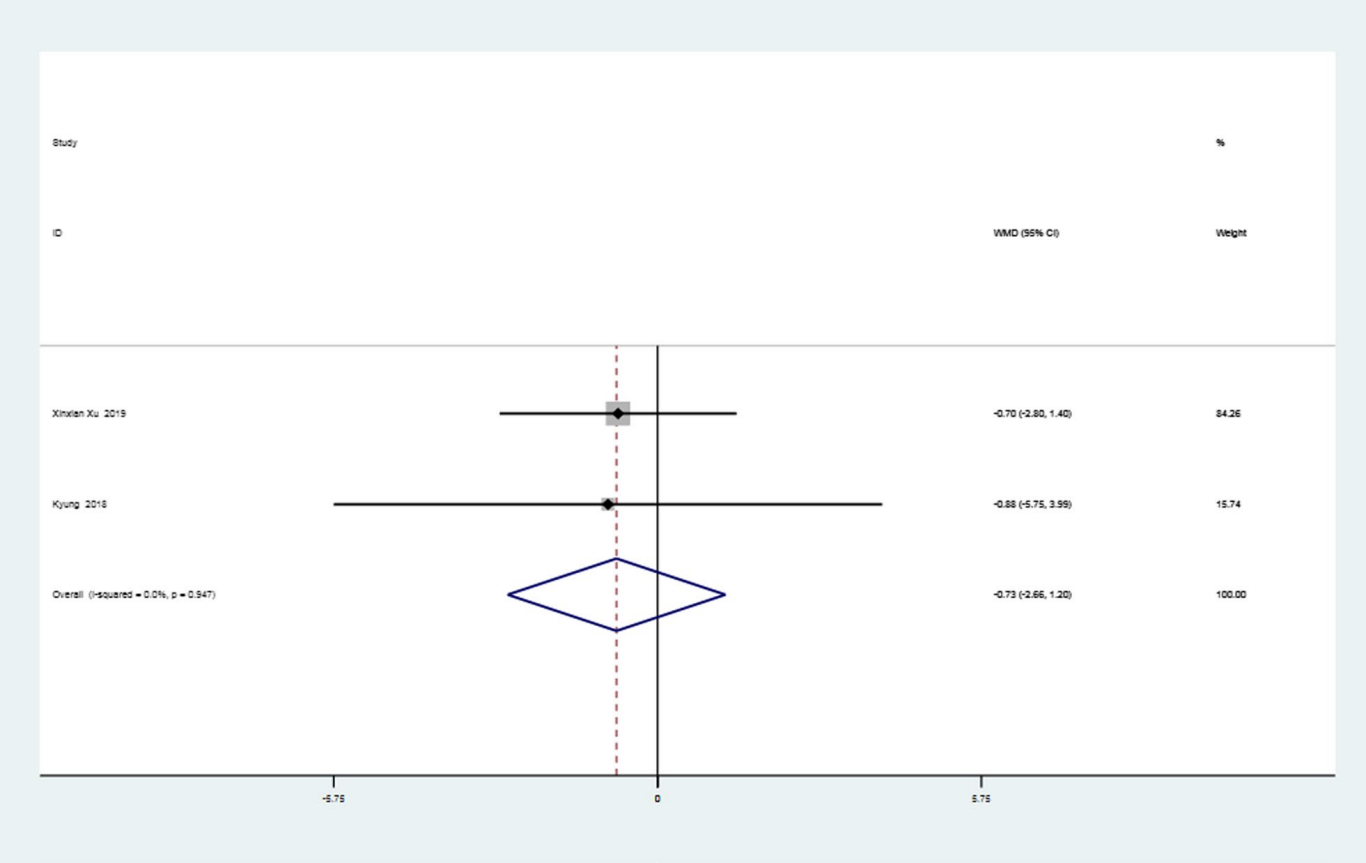
Forest plot of ASES score in the first year after surgery. *Note* WMD = mean value of knotted group—mean value of knotless group

The two studies [20, 37] that reported the ASES scores in the first year after surgery were subjected to a heterogeneity test, which revealed strong homogeneity in the studies (*I*^2^ = 0% < 50%, *P* = 0.95 > 0.1). The meta-analysis revealed that, after one year following surgery, the ASES score in the knotless group was 0.73 points higher than that in the knotted group, with the difference being statistically insignificant (MD =  − 0.73, 95%CI − 2.66 to 1.20, *P* = 0.46 > 0.05). The data are illustrated in Fig. 6.

### ASES score in the second year after surgery

**Fig. 7:**
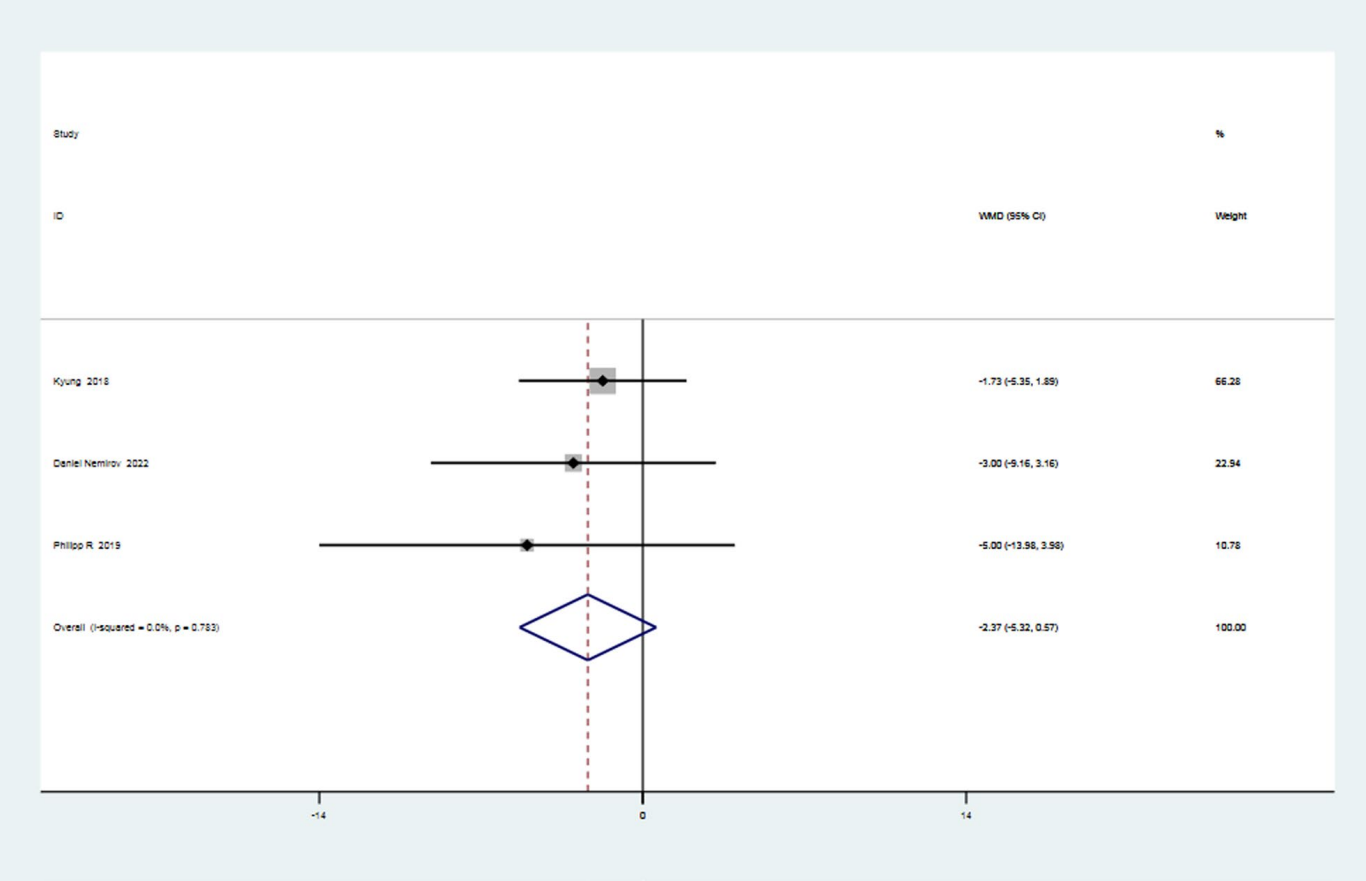
Forest plot of ASES score in the second year after surgery. *Note* WMD = mean value of knotted group—mean value of knotless group

The three studies [16, 20, 28] that reported the ASES scores in the second year after surgery underwent a heterogeneity test, which revealed that there was none (*I*^2^ = 0% < 50%, *P* = 0.78 > 0.1). The meta-analysis revealed that the ASES score in the knotless group was 2.37 points higher than that in the knotted group two years post-surgery, though the difference was statistically insignificant (MD =  − 2.37, 95%CI − 5.32 to 0.57, *P* = 0.12 > 0.05). Details are shown in Fig. 7.

### Constant score in the first year and in the second year after surgery

**Fig. 8:**
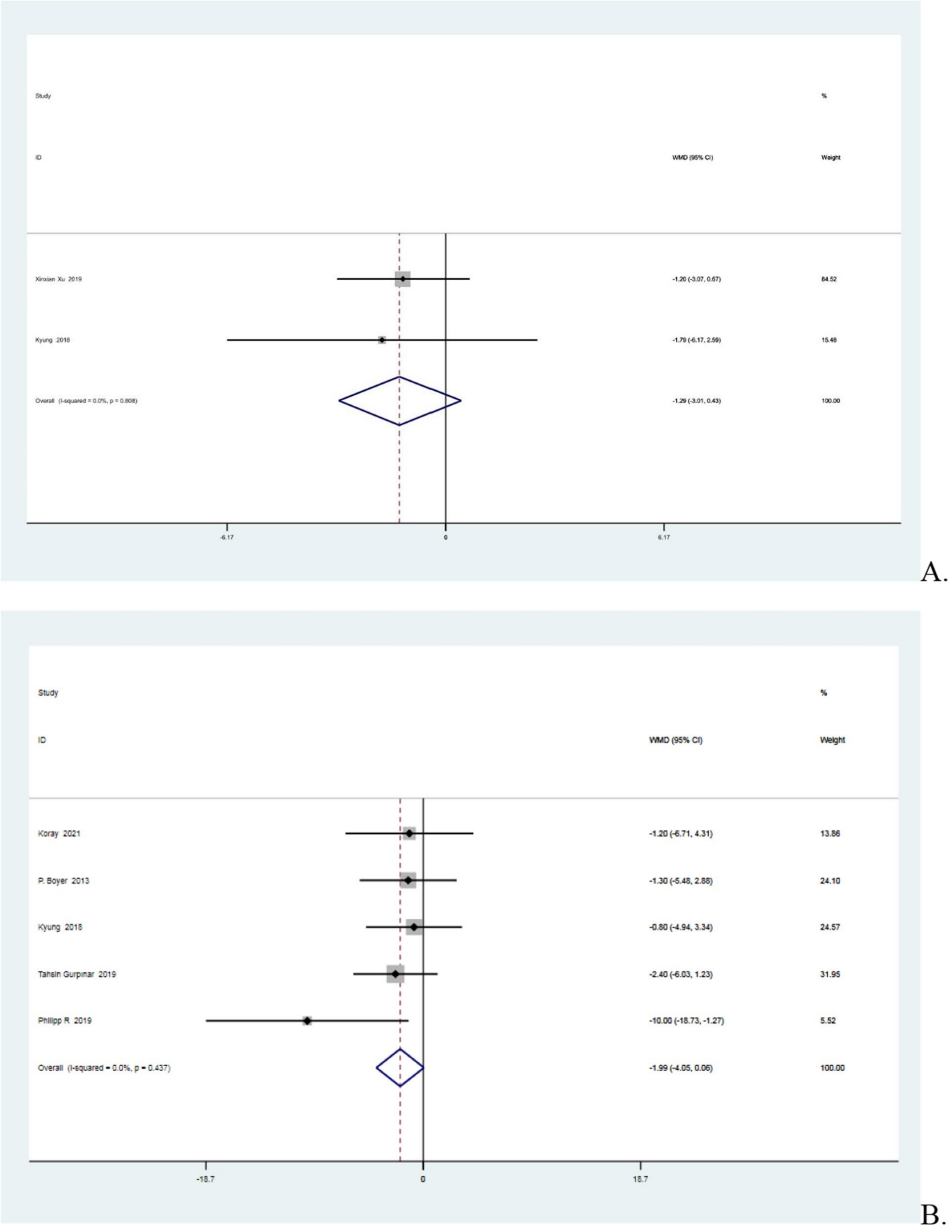
**A** Forest plot of Constant score in the first year after surgery. **B** Forest plot of Constant score in the second year after surgery. *Note* WMD = mean value of knotted group—mean value of knotless group

Two studies [20, 37] reported the Constant scores in the first year after surgery and five [3, 15, 16, 20, 34] reported the Constant scores in the second year after surgery. All seven studies were non-heterogeneous (*I*^2^ = 0% < 50%, *P* = 0.81 > 0.1 in the first year; *I*^2^ = 0% < 50%, *P* = 0.44 > 0.1 in the second year). The meta-analysis showed that the Constant score in the knotless group was 1.29 points higher than in the knotted group at the one-year mark and 1.99 points higher at the two-year mark. However, the differences were negligible (MD =  − 1.29, 95%CI − 3.01 to 0.43, *P* = 0.14 > 0.05 in the first year and MD =  − 1.99, 95%CI − 4.05 to 0.06, *P* = 0.06 > 0.05 in the second year). Details are shown in Fig. 8.

### Postoperative forward flexion mobility

**Fig. 9:**
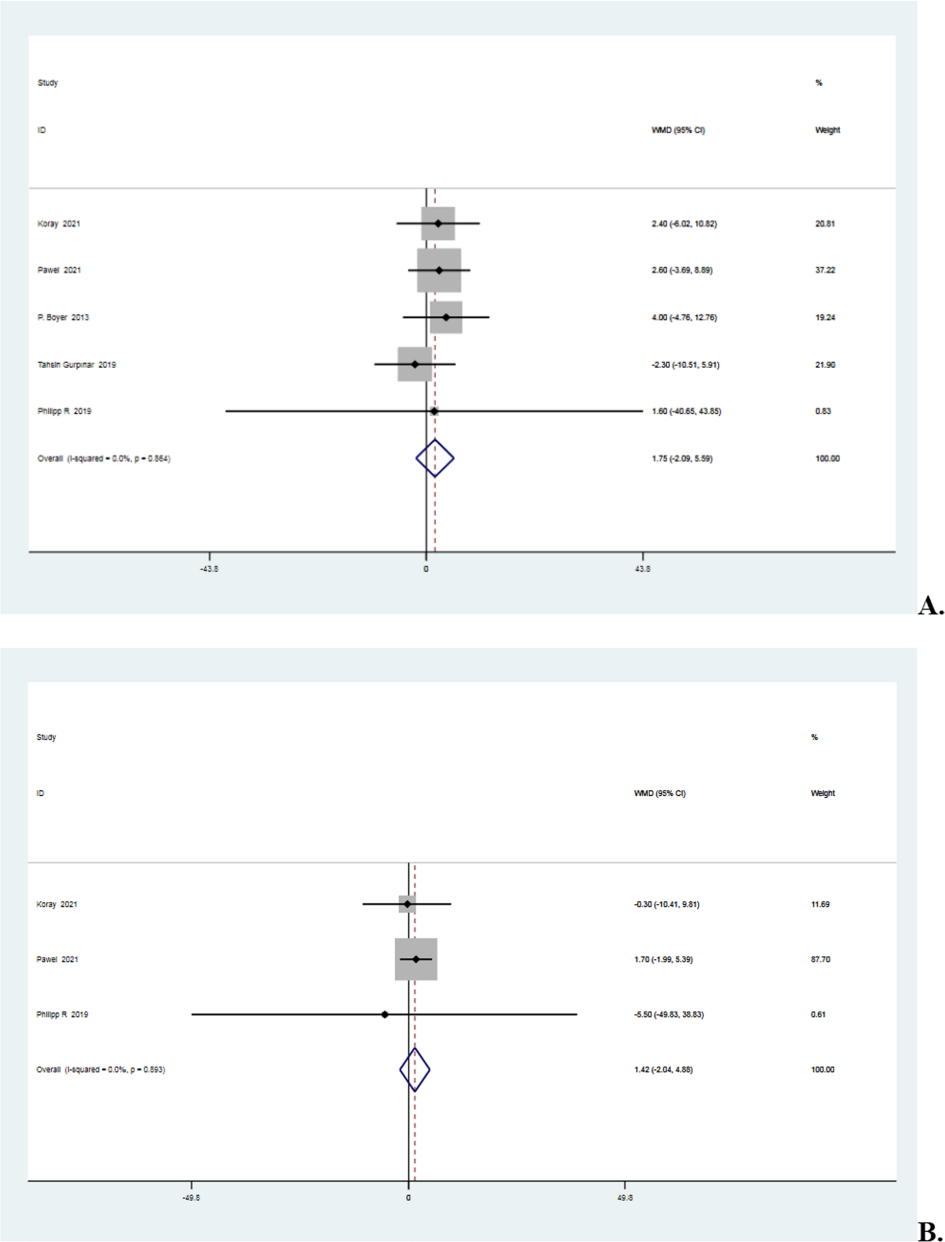

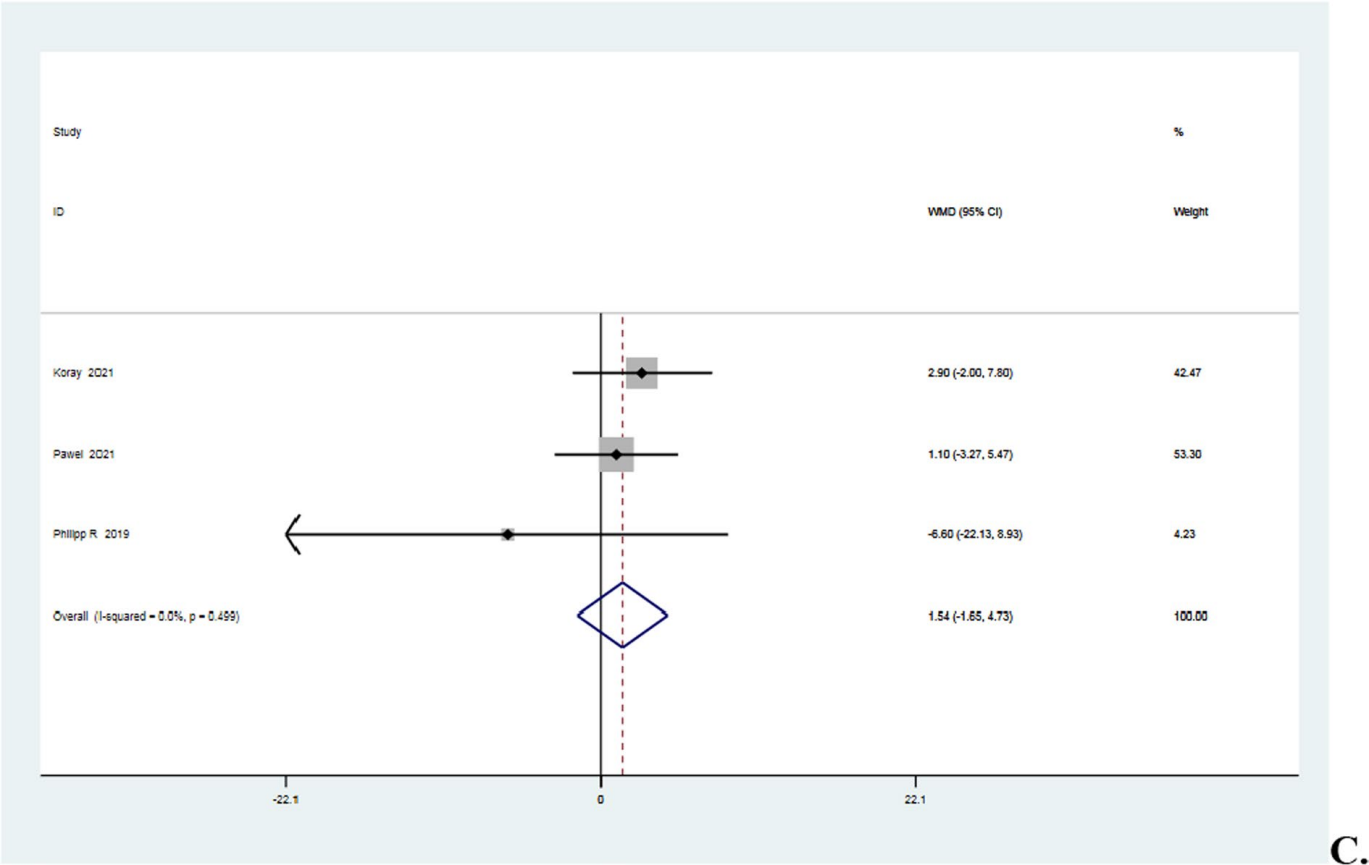
**A** Forest plot of Postoperative Forward Flexion Mobility. **B** Forest plot of postoperative abduction mobility. **C** Forest plot of external rotation mobility. *Note* WMD = mean value of knotted group—mean value of knotless group

Variation was lacking in the five studies [3, 15, 16, 34, 40] that reported postoperative forward flexion mobility (*I*^2^ = 0% < 50%, *P* = 0.86 > 0.1). The meta-analysis indicated that the knotted group had 1.75 degrees more forward flexion mobility than the knotless group, though the difference was statistically insignificant (MD = 1.75, 95%CI − 2.09 to 5.59, *P* = 0.37 > 0.05). Details are shown in Fig. 9A.

### Postoperative abduction mobility and external rotation mobility

The three studies [16, 34, 40] that reported postoperative external rotation and abduction mobility were also considerably homogeneous (*I*^2^ = 0% < 50%, *P* = 0.89 > 0.1 in abduction part and *I*^2^ = 0% < 50%, *P* = 0.50 > 0.1 in external rotation part). The meta-analysis revealed that the knotted group had 1.42 degrees more forward flexion mobility and 1.54 degrees more external rotation mobility than the knotless group, even though the difference was statistically insignificant (MD = 1.42, 95%CI − 2.04 to 4.88, *P* = 0.42 > 0.05 in terms of abduction and MD = 1.54, 95%CI − 1.65 to 4.73, *P* = 0.35 > 0.05 in terms of external rotation). Details are shown in Fig. 9B, C.

### Postoperative pain score

**Fig. 10:**
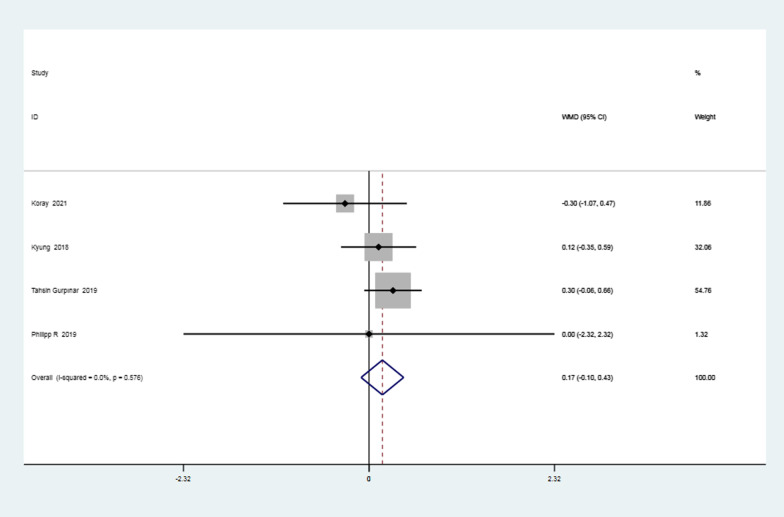
Forest plot of postoperative pain score. *Note* WMD = mean value of knotted group—mean value of knotless group

The four studies [15, 16, 20, 34] that reported postoperative VAS scores were homogeneous (*I*^2^ = 0% < 50%, *P* = 0.58 > 0.1). The meta-analysis revealed that the knotted group's pain score was 0.17 points higher than the knotless group's, though the difference was statistically insignificant (MD = 0.17, 95%CI − 0.10 to 0.43, *P* = 0.22 > 0.05). Details are shown in Fig. 10.

### Publication bias test

**Fig. 11:**
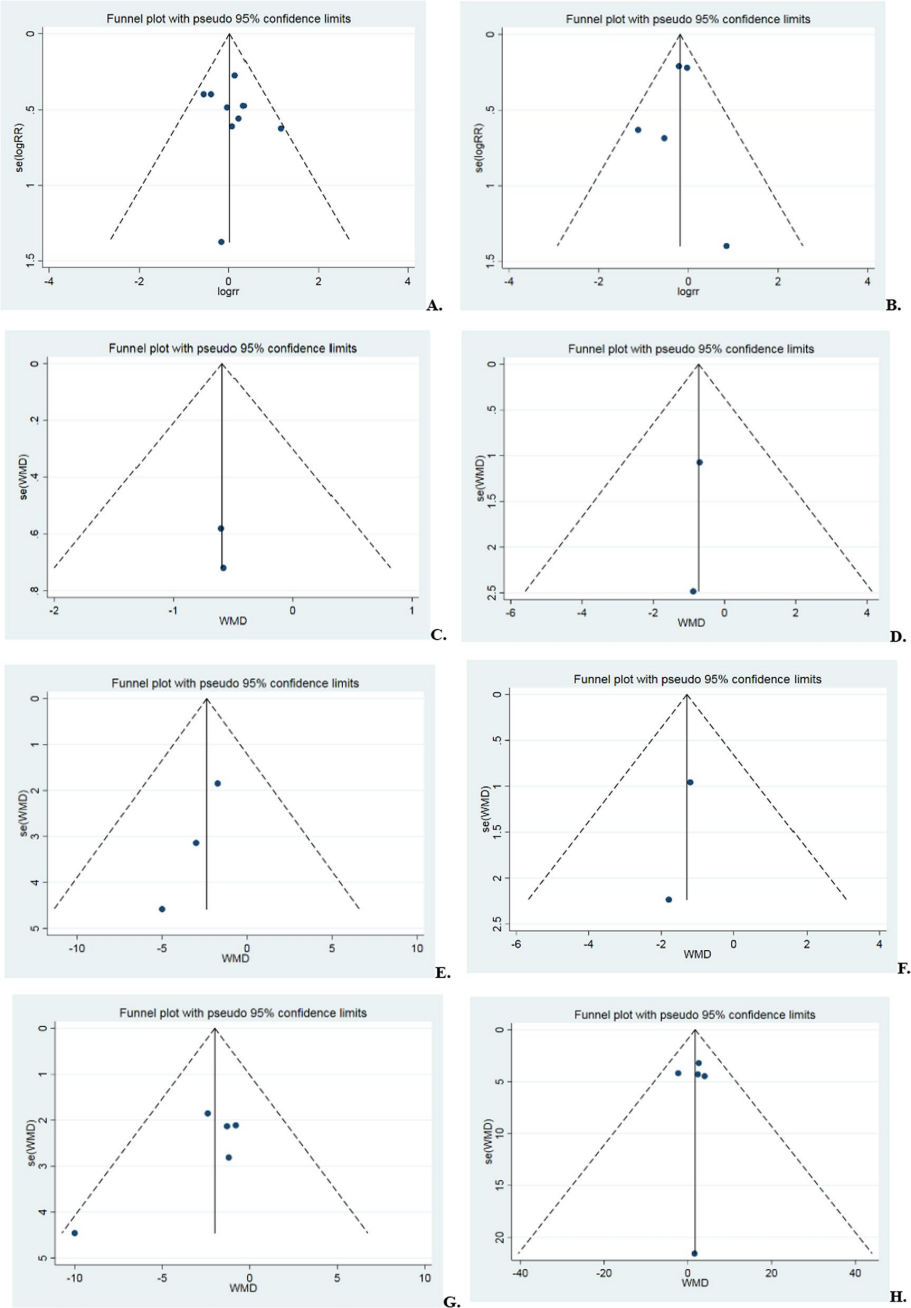

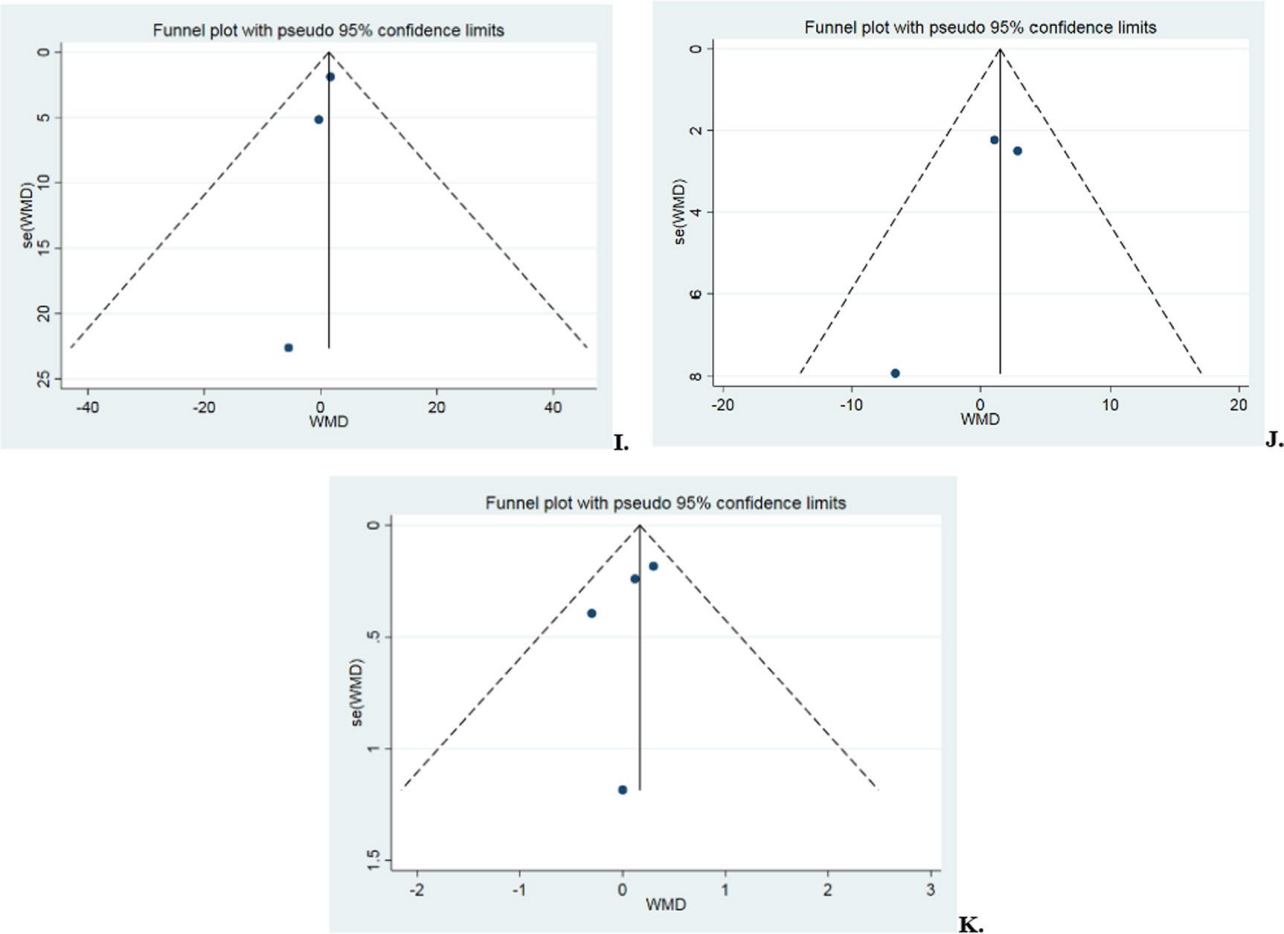
Funnel plot of the 11 results. (**A** postoperative retear rate, **B** postoperative retear classification, **C** UCLA scores in the first year postoperatively, **D** ASES scores in the first year postoperatively, **E** ASES scores in the second year postoperatively, **F** Constant scores in the first year postoperatively, **G** Constant scores in the second year postoperatively, **H** postoperative anterior flexion mobility,** I** postoperative abduction mobility, **J** postoperative external rotation mobility, **K** postoperative pain scores)

The test of Begg’s was used for the publication bias test in the above outcomes. The findings indicated a lack of correlation, deeming the publications unbiased. Details are shown in Fig. 11 and Table 4.

**Table 4:** Begg’s test for publication bias of pairwise meta-analysis

Outcome	Postoperative retear rate	Postoperative retear classification	UCLA score in the first year after surgery	ASES score in the first year after surgery	ASES score in the second year after surgery	Constant score in the first year after surgery	Constant score in the second year after surgery	Postoperative Forward flexion mobility	Postoperative abduction mobility	Postoperative external rotation mobility	Postoperative pain score
*P*	0.75	1.00	1.00	1.00	0.30	1.00	0.46	1.00	1.00	1.00	0.73

Low publication bias: *P* > 0.05

*UCLA* University of California, Los Angeles, *ASES* American Shoulder and Elbow Surgeons

## Discussion

In this meta-analysis, there were 10 original articles on the clinical outcomes of double-row suture bridge sutures for rotator cuff injuries, both with and without knots in the medial row, including 1 randomized controlled trial, 4 prospective cohort studies, and 5 retrospective cohort studies. The meta-analysis was focused on postoperative retear, postoperative retear classification, postoperative shoulder function score, postoperative shoulder mobility, and postoperative pain, respectively.

The results showed slight differences between the two techniques, namely, a slightly higher postoperative retear rate in the knotted group than in the knotless group (*P* = 0.74), a higher percentage of postoperative type 2 retear in the knotted group, and a higher proportion of type 1 retear in the knotless group (*P* = 0.14). In terms of postoperative functional scores, the results showed better clinical outcomes in the knotless group than in the knotted group (For details please see Figs. 6, 7 and 8). As for postoperative pain, the knotless group had lower pain scores (*P* = 0.22), but none of the above differences were statistically significant.

### Clinical implications and implications for future research

The double-row suture bridge technique is widely preferred by orthopedic surgeons globally because of the improved contact area between the rotator cuff and bone, increased yield load, and reduced operative time and cost [15, 19, 22, 24, 27].

The debate over whether or not to tie the medial row of sutures used in a rotator cuff suture bridge repair has gained increased interest. The most important indicator for the assessment of its clinical outcomes is the tendon retear rate. The effectiveness of the suture bridge technique with medial row tying has been reported to be comparable to that of the approach with no tying in many therapeutically relevant trials, including postoperative tendon retear rate, functional scores, and pain score outcomes [17, 34, 37]. The findings of this meta-analysis are consistent with the results of the aforementioned studies.

In spite of the high level of scientific evidence supporting use of knotted suture bridges, a few studies have suggested that the use of this technique is linked to significant retear rates, particularly type 2 repair failure [6, 38]. Trantalis and his colleague [36] reported that type 2 failure was linked to excessive tension between the tendon and suture at the medial row and overtightening of the medial knots. Cho et al. [5] noted that sutures may cause vascular strangulation around the tendon, bringing about tendon necrosis. Vascularity around the rotator cuff was a key determinant of the effectiveness of the surgery. A practical Doppler flowmetry measurement of intraoperative blood flow showed that the intratendinous blood flow significantly decreased with the use of knotted suture bridge technique rotator cuff repair after placing lateral-row anchors. The impact of the knotless on circulation around the site was, however, not evaluated in this review [7]. The above studies suggested that suture bridges with knotless medial rows may facilitate faster healing of the tendon and reduce the retear rate compared to knotted medial rows. A potential biomechanical advantage of knotless constructs is “self-reinforcement”, which is a mechanism where increasing the tensile load can generate increased resistance to structural failure overtime [30]. Yong et al. [33] reported that the knotless group had a considerably lower retear rate relative to the conventional knot-tying group, which is consistent with the above points. However, due to an insufficient sample sizes, the scientific evidence was not adequate enough to support generalized use of this technique. Therefore, it requires careful assessment and consideration for patients with shoulder osteoporosis before rotator cuff suturing.

The suture anchor constructed using the knotless suture bridge procedure may fail because stress is typically centered there rather than at the medial row, especially in patients suffering from osteoporosis. Ultimately, without adequate compression by the medial row suture limbs, the healing of a repaired rotator cuff may be hampered. Therefore, suture repair of the rotator cuff in patients with osteoporosis requires careful assessment and consideration on the surgeons’ part [21, 23].

This meta-analysis revealed that postoperative tendon retear rates among patients for whom the knotted medial row suture bridge method was used did not significantly contrast with rates among those for whom the knotless suture bridge approach was used. Therefore, clinicians should be wary of the occurrence of postoperative rotator cuff retear during patient recovery, regardless of whether the knotted or knotless medial row of the suture bridge was used.

In a study by Galatz and her colleagues [13], patients with arthroscopic repair of large and massive rotator cuff tears may have higher postoperative function and better satisfaction, but the results may mask underlying failures in healing. Chung et al. [8] found that 39.8% of patients in arthroscopically repaired massive rotator cuff tears had better postoperative functional recovery but rotator cuff anatomy healed incompletely. Clinical outcomes after rotator cuff suturing may also be influenced by the following factors: diabetes, wound infection, surgical experience, suture material, and inappropriate postoperative rehabilitation [39]. If the rehabilitation is too aggressive, it may cause the suture to fail, thus increasing the rate of tendon retear. The effects of these confounding factors should be investigated in future research.

## Limitations

The article is also associated with a few limitations. Firstly, the tendon tear size and retraction were not considered in this analysis, which may affect the rotator cuff retear rate. It is likely that some articles were not mentioned or had less relevant data in the analysis. Secondly, the size of the pre-suture tear may affect the retear rate. Some of the original articles did not restrict the patient's preoperative tear type such as Şahin et al. [34] trial. Some articles strictly restricted the type of tendon tear before suture, for example, Yong et al. [33] restricted patients to medium-sized tears of the tendon, and they excluded small, large, and massive tears. Besides, there was no significant difference between the experimental and control groups at baseline in each article, so we did not take it into account. In addition, there was variation in the surgical procedures included in the analysis. Other than rotator cuff repair, other procedures such as acromioplasty and glenoid labrum repair may be involved. One RCT and nine cohort studies were included in this paper, which may have an impact on the results in terms of study methodology, and more high-quality studies with large samples are needed to reduce this heterogeneity in the future. Besides, tendon retraction and fat infiltration after rotator cuff tears also significantly influenced postoperative retear rates, with no significant difference between the knotted and knotless groups in the original literatures. In other words, there was no significant difference in the effect on retear rates. This study focused on retear rates, as baseline data for tendon retraction and fat infiltration after rotator cuff tears were not significantly different in each article, and therefore these two indicators were not included in the analysis.

## Conclusion

The clinical outcomes of shoulder arthroscopic rotator cuff repair with the suture bridge technique with or without a knotted medial row was proven to be equivalent. These outcomes are about postoperative retear, postoperative retear classification, postoperative shoulder function score, postoperative shoulder mobility, and postoperative pain, respectively. It should be noted that the conclusions are based on short-term clinical follow-up data.

## Data Availability

For further enquiries about the relevant original materials of this article, please consult the corresponding author.

## Abbreviations

NOS: Newcastle–Ottawa scale
RCT: Randomized controlled trial
UCLA: University of California, Los Angeles
ASES: American Shoulder and Elbow Surgeons
SSI: Shoulder strength index
CI: Confidence interval
RR: Risk ratio
MD: Mean difference
M ± SD: Mean ± standard deviation
